# Trace of evanescent wave polarization by atomic vapor spectroscopy

**DOI:** 10.1038/s41598-021-01264-9

**Published:** 2021-11-04

**Authors:** M. Mosleh, M. Ranjbaran, S. M. Hamidi

**Affiliations:** 1 Magneto-Plasmonic Lab, Laser and Plasma Research Institute, Shahid Beheshti University, Tehran, Iran; 2 Department of Physics, Central Tehran Branch, Islamic Azad University, Tehran, Iran

**Keywords:** Applied optics, Optical techniques, Optical spectroscopy

## Abstract

Various efforts have been made to determine the polarization state of evanescent waves in different structures. The present study shows the reliability of magneto-optical spectroscopy of D1 and D2 lines of rubidium metal and polarization-dependent transitions to investigate and trace the changes in the polarization state of evanescent fields during total internal reflection over different angles. For this purpose, we design and fabricate atomic- evanescent Rb vapor cells and examine the effect of polarization changes of evanescent waves, depending on the propagation direction of evanescent waves in anisotropic rubidium vapor media under 88 mT external magnetic field by different configurations theoretically and experimentally. The results confirm the dependency of allowed $\sigma^{ \pm } { }\;{\text{and}}\;\pi$ transitions on the magneto optical configuration as a tool to determine changes in the polarization of evanescent waves in more complicated wave states in anisotropic media.

## Introduction

The interaction of alkali metal atoms with light is the cornerstone of valuable applications such as atomic magnetometers, atomic clocks, and miniaturized atomic sensors^1,2^. These miniaturized atomic sensors can enhance the spatial resolution by reducing the size by manipulating the optical field in low dimensions via near-field modes^3–7^. These near-field modes in nanophotonics, named evanescent fields, can be achieved via several techniques, such as total internal reflection in prisms, grating or perforated nanostructures^8^, waveguides^9^ and tapered fibers^10^. Exact knowledge of the polarization state of evanescent waves would be as a crucial tool in nanophotonic localization applications, such as magnetic memories, characterization of the chiral state of biological molecules and all-optical magnetic recording in the submicron dimension using circular polarization of the near field of plasmonic structures^11^. Even neurons of living types respond to polarization of light in some cases; for example, specific types of insects can navigate with polarization of skylight^12^. In addition, advanced quantum technologies inspired studying atom light interactions by evanescent waves of nanostructures. Evanescent field trapping of atoms by surface plasmon resonance shows that the polarization state of optical modes plays a crucial role in different fundamental light-matter interactions^13^. Additionally, cooled atom trapping using tapered nanofibers directly shows the impact of the polarization state of the electric field of the evanescent wave surrounding the fiber and the advantage of controlling the polarization state of the evanescent wave as a tool to adjust the trapping position around the fiber^14,15^. Also in the field of quantum information and metrology, evanescent electromagnetically induced transparency (EIT) plays important role^16^.

Complete control of the interaction of the evanescent field and atomic vapor and its investigation require a deep understanding of both evanescent wave and atomic gas ensemble characteristics. Optical fields carry momentum and spin, which depend on the field wave vector and polarization, respectively, and there is more sense for free space optical fields despite evanescent wave cases^17^. One of the main differences is that the spin of evanescent fields is orthogonal to the wave vector, despite free space fields where spin and wave vectors are parallel^18^. Additionally, because of the nonparallel directions of real and imaginary parts of the wave vector, the evanescent wave is known as an inhomogeneous wave^19^. Numerous publications have shown the design of nanostructures with the purpose of exciting specific polarizations in nanostructures^20^ in simulation ways. It is well known that the production of nanostructures is not as ideal as expected in simulations, and there are always small differences between the produced nanostructures and the predicted nanostructures. Therefore, the existence of a practical method to measure the polarization state of evanescent fields would be necessary in advanced works.

Recent investigations have revealed efforts to experimentally measure the complex and extraordinary properties of polarization of evanescent fields in nanostructures^21,22^. There are known methods to experimentally investigate the theoretical results of evanescent wave characterization. One of them is based on Mie scattering of evanescent waves from isotropic spherical particles located at the interface of glass prisms and water media^23^. Although these studies provide an accurate experimental demonstration of the inherent properties of evanescent waves, a special condition of experiments makes this type of study out of reach for many researchers^24^. Experimental demonstration of the polarization state of the evanescent field in different apertures like the tips of scanning near field microscopy (SNOM) is important in applications such as the observation of the magnetic domain of materials in nm size. Reported methods are based on far-field scattered light detection from the nearfield area between tips and nanometric samples. This method faces different problems, such as reference scattering from the background and unequal far-field leakage scattering of different near-field modes^25,26^.

Other experiments based on atomic spectroscopy show valuable potential to reveal the polarization state of evanescent waves in Zeeman effect observations^27^. They showed interaction of evanescent wave with atoms is sensitive to external magnetic field and applied different configurations of light and magnetic field to observe the contributions of π and σ Zeeman split lines on absorption spectra.

In addition to interest in studying evanescent waves, laser atomic spectroscopy can open up the way to follow the changes in properties of atoms near the surface, such as changes in the transition probability of quadrupolar transitions of atoms near solid surfaces^28^. Knowledge of the properties of both evanescent waves and atoms near the surfaces allows engineering of hyperfine spectra of atoms as well as the possibility of creating miniaturized quantum sensors as small as metallic tips of scanning near filed optical microscopy microscopes^29^.

The propagation of abnormal evanescent fields in atomic media is completely dependent on the characteristics of dispersive atomic vapor and is described through susceptibility. In the case there is no external magnetic field applied to the vapor medium, the susceptibility of atomic gas would be calculated by taking into account hyperfine splitting, the thermal motion of atoms, the oscillator strength of allowed transitions, and broadenings of different kinds of atoms like as rubidium and sodium^30,31^. The ensemble of atomic gas under an external magnetic field acts as an anisotropic optical medium, and the propagation of light on it would be described by the susceptibility tensor of atoms. Alternatively, polarization is a fundamental property of electromagnetic fields controlling the light matter interaction.

The role of anisotropy of atomic vapor on polarization spectroscopy of evanescent wave and the possibility to trace any change on waves' polarization through tracing selected atomic transitions signal is the subject of our theoretical simulations and experimental measurements. We compare experimentally measured spectra of an unknown polarized evanescent field with simulated spectra of certain polarizations to show the sensitivity of atomic spectroscopy to changes in polarization. Provided simulations are based on calculation of the magnetooptical polarizability tensor as well as normal mode analysis using Maxwell equation.

## Evanescent wave absorption: calculation

**Figure 1:**
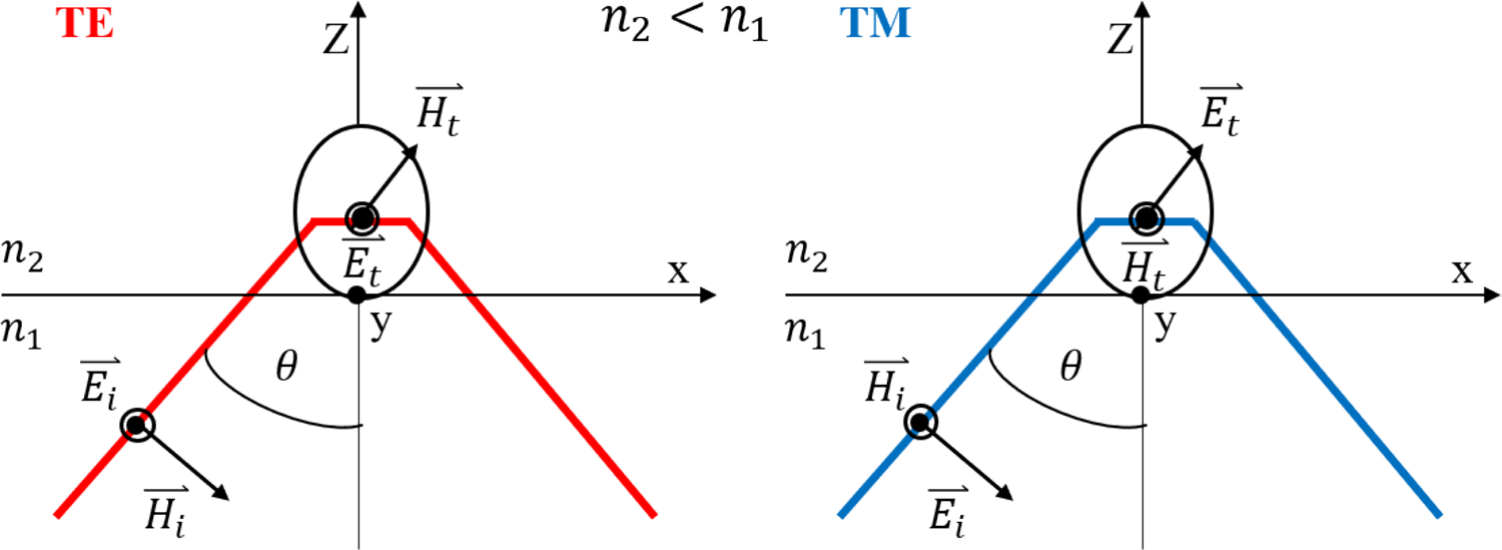
Polarization state of evanescent waves at total internal reflection for TE and TM waves.

In this study, excitation of evanescent waves was performed through total internal reflection of laser light from the prism-Rb vapor interface. We measure the reflection from the interface of a triangular prism and Rb atomic vapor in the presence of an external magnetic field, which is mainly dependent on the polarizability response of the atomic gas media and the properties of the evanescent wave, primarily the polarization of the evanescent wave. The polarization state of evanescent waves depends on the incident angle and the polarization of incoming laser light. In the case of TE(s) and TM(p) incoming polarizations, the state of evanescent wave polarization remains linear and changes to elliptical (Fig. 1). The electric field components of the evanescent wave in a dilute Rb vapor medium can be calculated by Fresnel equations in the case of these polarizations^32,33^. For the case of s and p polarization,

1 $$ \begin{aligned} E_{x} & = \frac{{(2\cos \theta )(\sin^{2} \theta - n^{2} )^{\frac{1}{2}} }}{{(n^{4} \cos^{2} \theta + \sin^{2} \theta - n^{2} )}}E_{p}^{i} \exp \left( { - i(\delta_{p} + \frac{\pi }{2})} \right) \\ E_{z} & = \frac{{2\cos \theta \sin^{2} \theta }}{{(n^{4} \cos^{2} \theta + \sin^{2} \theta - n^{2} )}}E_{p}^{i} \exp ( - i\delta_{p} ){,}\quad \delta_{p} = \tan^{ - 1} \left( {\frac{{(\sin^{2} \theta - n^{2} )^{\frac{1}{2}} }}{{n^{2} \cos \theta }}} \right) \\ E_{y} = & \frac{2\cos \theta }{{(1 - n^{2} )^{\frac{1}{2}} }}E_{s}^{i} \exp \left( { - i\frac{{\delta_{s} }}{2}} \right){,}\quad \delta_{s} = \frac{{(\sin^{2} \theta - n^{2} )^{2} }}{\cos \theta } \\ \end{aligned} $$

where θ is the angle of incidence, $$n$$ denotes the refractive index of the prism, $$E_{x}$$ and $$E_{z}$$ represent the components of the polarization ellipse in the case of incoming TM polarization, and $$E_{y}$$ shows the electric field component of incoming TE polarization. It is obvious from the equations of TM polarization that there is a $${\raise0.7ex\hbox{$\pi $} \!\mathord{\left/ {\vphantom {\pi 2}}\right.\kern-\nulldelimiterspace} \!\lower0.7ex\hbox{$2$}}$$ phase difference between the two parameters causing the linear p-polarization to be converted to elliptical polarization. To achieve the best insight into the interaction of evanescent waves and atomic media on reflection, our approach considers the evanescent wave as an independent wave and models its propagation in an anisotropic medium based on available contents on the propagation of polarized light in atomic vapor. Figure 1 shows that incoming light after conversion to an evanescent field propagates some distance in the second medium and interacts with Rb atoms. The energy of the evanescent field couples to atomic transitions at frequencies equal to atomic transitions and will be eliminated from the reflected optical power. In the following, we will show how to characterize the properties of Rb atomic gas under the applied magnetic field.

The atomic vapor polarizability tensor becomes nondiagonal due to its anisotropic behavior. For instance, it is well known that by applying an external magnetic field in the direction of the z axis, the polarizability tensor can be written as a function of dielectric elements ε and gyration factor g^34^.

To forecast the polarization state of transmitted or reflected light, we need to decompose any arbitrary polarization of incoming light based on normal modes. Normal modes of propagation of light in the anisotropic medium are the results of Maxwell equations. Supposing laser light as a plane wave and its dependence on time and coordinates, the main equation of light propagation will be derived from Maxwell equations as

2 $$ n^{2} E - n(n.E) = \varepsilon E $$

where $n = \left( {\frac{c}{\omega }} \right)k$ is the refraction vector. This equation has solutions only if its determinant is equal to zero,

3 $$ \det \left\| {n^{2} \delta_{ik} - n_{i} n_{k} - \varepsilon_{ik} (\omega )} \right\| = 0 $$

Substituting the results of the derived refraction indices into Eq. (2) will give eigenvectors belonging to each appropriate refractive index. This means that different polarizations of light will experience different refractive indices. The polarizability tensor can be diagonalized based on the abovementioned eigenvectors of polarization as^35^;

4 $$ \left[ {\begin{array}{*{20}c} {\varepsilon_{x} - ig} & 0 & 0 \\ 0 & {\varepsilon_{x} - ig} & 0 \\ 0 & 0 & {\varepsilon_{z} } \\ \end{array} } \right] = \varepsilon_{0} + \varepsilon_{0} \left[ {\begin{array}{*{20}c} {\chi_{ + } } & 0 & 0 \\ 0 & {\chi_{ - } } & 0 \\ 0 & 0 & {\chi_{0} } \\ \end{array} } \right] $$

where $\chi_{ \pm }$ and $\chi_{0}$ are polarizabilities related to different circular and linear polarizations, respectively. This allows us to relate the elements of the polarizability tensor to the susceptibility of atomic transitions with the possible degrees for changes in angular momentum as $\pm 1{ }$ and 0.

5 $$ \varepsilon_{x} = \frac{{\varepsilon_{0} }}{2}(2 + \chi_{ + } + \chi_{ - } )\quad g = \frac{{\varepsilon_{0} }}{2}i(\chi_{ - } + \chi_{ + } )\quad \, \varepsilon_{z} = \varepsilon_{0} (1 + \chi_{0} ) $$

Having this polarizability tensor and its three main elements in hand allows predicting the light propagation in the vapor medium for arbitrarily applied magnetic fields in Faraday or Voigt configurations.

Alternatively, considering the population of atoms in any state at different temperatures and Doppler broadening in response to thermal motion, the polarizability of atoms can be written as^36^.

6 $$ \begin{aligned} \chi_{q} (\nu ) & = \frac{{iN_{0} }}{{\varepsilon_{0} h\nu_{0} }}\sqrt {\frac{{\pi M_{0} c^{2} }}{2kT}} \sum\limits_{{MM^{^{\prime}} }} {\left[ {B_{M} S_{q} (M,M^{^{\prime}} )W(\xi )} \right]} \\ \xi & = \sqrt {\frac{{\pi M_{0} c^{2} }}{2kT}} \left( {\frac{{\nu_{0} - \nu - \Delta \nu_{{M,M^{^{\prime}} }} }}{{\nu_{0} }} + \frac{i}{{4\pi \nu_{0} \tau }}} \right) \\ \end{aligned} $$

where $N_{0}$ is the total number density, $B_{M}$ denotes the Boltzmann distribution for level |M > and is given by

7 $$ B_{M} = \frac{{e^{{ - \frac{{h\Delta \nu_{M} }}{kT}}} }}{{\sum {Me^{{ - \frac{{h\Delta \nu_{M} }}{kT}}} } }} $$

$q = \Delta M = \pm 1,{ }0$, $S_{q} \left( {M,M^{\prime}} \right)$ is the Zeeman line strength, M and $M^{\prime}$ represent the magnetic quantum numbers of the ground and excited states, $\Delta \nu_{M}$ is the energy level shift relative to the center of gravity $\nu_{0}$, and W denotes the plasma distribution function. The summation includes all single transitions. Note that in the calculation above, the intensity of laser light is supposed to be very low, so optical pumping is neglected, and the effect of neighboring transitions on each other is zero. We can add the polarizability of all single transitions to make the total polarizability tensor.

New transition frequencies and probabilities because of Zeeman splitting and transitions originating from the dependent Hamiltonian in the presence of an external magnetic field are determined as follows:

8 $$ H = H_{0} + H_{z} $$

where $H_{0}$ includes fine and hyperfine interaction energy levels and $H_{z}$ represents Zeeman splitting of atoms. The matrix elements of this Hamiltonian and the strength of the interaction of light and atoms in a specific transition can be calculated by the eigenvalue equation and dipole matrix element based on the transition eigenstate vectors, as fully described in^34^. In this simulation, the transition strength related to the transition probability is calculated by $\left| {\left\langle {g\left| d \right|e} \right\rangle } \right|^{2}$, where d is the transition dipole and $\left\langle g \right|$ $\left| e \right\rangle$ represents the ground plus excited states^37,38^. The effect of the applied magnetic field would be understood by considering Wigner 3-j and 6-j symbols in this equation, constituting the essence of geometrical symmetry of the system^39^. Magnetic field-induced anisotropy is a result of symmetry breaking in the system and appears as a change in Clebsch-Gordon coefficients in response to changes in the Wigner 3-j and 6-j symbols.

Using these equations and the polarization of incoming light onto Rb vapor as our atomic media, simulations of absorption caused by the propagation of polarized electromagnetic fields in anisotropic Rb atomic vapor were performed utilizing an open-source python-based program called Elecsus^40^. This program calculates the susceptibility and absorption of alkali metal vapor under an applied external magnetic field at a given temperature with the possibility of changing the direction of the electric field relative to the magnetic field in Faraday or Voigt configurations. To obtain the susceptibility and absorption spectrum related to each magneto optical configuration, the program calculates the associated transition strengths of $\sigma^{ + }$, $\sigma^{ - }$ or $\pi$($\Delta m = + 1, - 1{\text{ or }}0$ respectively) transitions based on the Hamiltonian in^38^ for each D-line. The main limitation of this program to simulate the complex polarization of evanescent waves is that Elecsus is designed for free space TEM light.

One of the features of evanescent waves is the possibility of acquiring elliptical-like polarization, meaning that the plane of polarization would be parallel to the direction of propagation. This is obviously in contrast to TEM propagating light in free space. Considering this important fact, we used simulations only for evanescent waves formed at the critical angle. In the next section, we present the experimentally measured data and compare the results to conclude the polarization state of evanescent waves.

## Evanescent wave absorption: measurement

**Figure 2:**
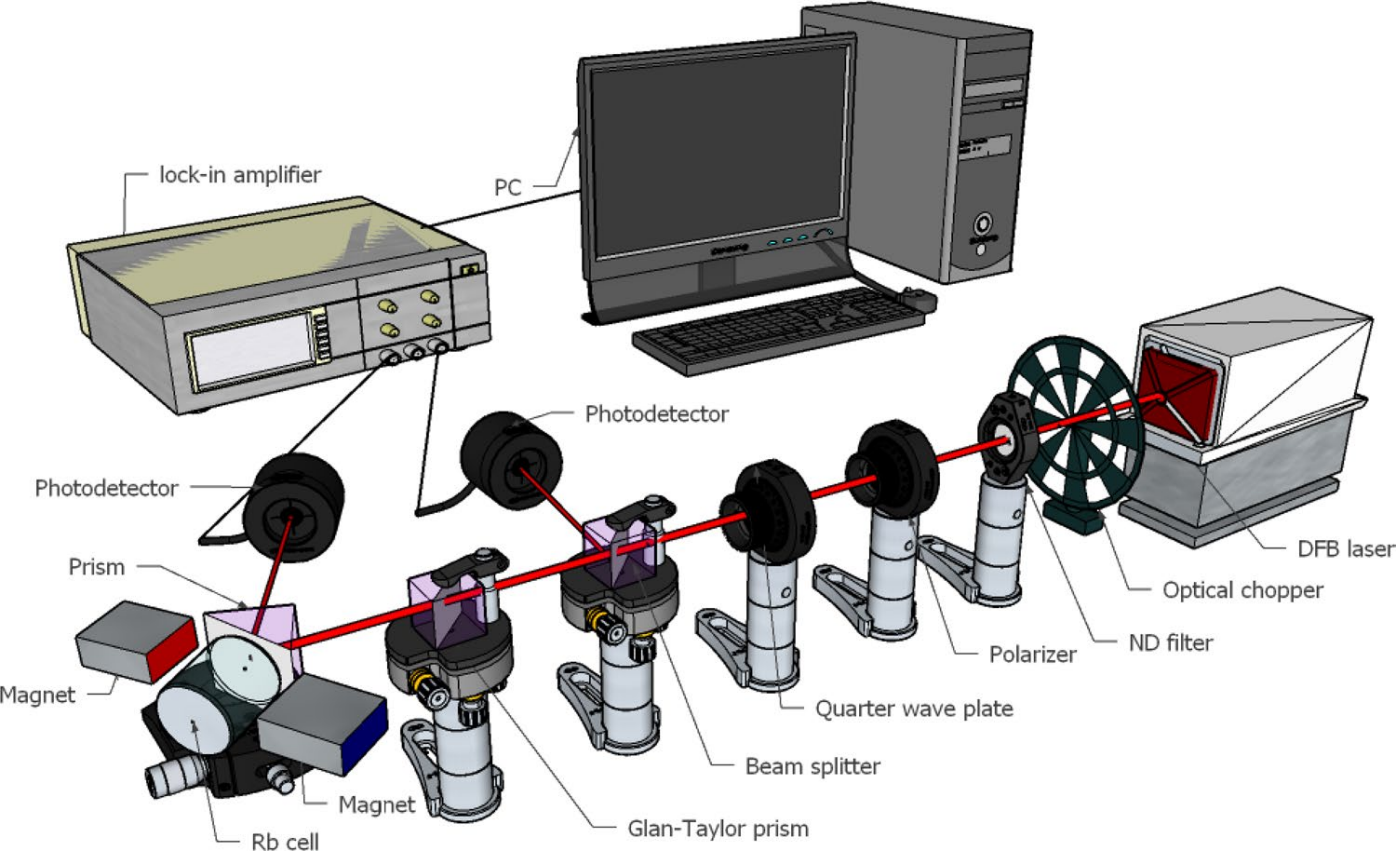
Optical setup used for magneto optical evanescent wave spectroscopy in the Faraday configuration.

Evanescent wave spectroscopy was performed by two distinct measurement setups, as shown in Fig. 2, under illumination with 795 nm and 780 nm tunable DFB lasers (Eagleyard DFB laser) with a 2 MHz linewidth. Linear or circular polarization of light was produced via a Glan-Taylor prism or set of polaroid and quarter wave plates, respectively. To obtain circular polarizations, we set the polarizer in 45° and then − 45° relative to the fast axis of the quarter wave plate. In final approve state, we use another polarizer to get sure about right or left circularly polarized light in the experimental part. To increase the signal-to-noise ratio (SNR), amplitude modulation was performed using an optical chopper. A beam splitter was used to divide the laser beam into the main and reference lines.

We produced a vapor cell to support the direct coupling of evanescent waves with Rb atomic vapor. A prism was epoxy glued to a Pyrex reservoir and evacuated, after which a droplet of natural Rb metal was inserted into the reservoir in the presence of 10^–4^ mTorr of argon gas, and the reservoir was terminated by a glass blowing technique. Rb metal is in the solid-state at room temperature, so we heated the cell to make a dense vapor of Rb atoms at approximately 150 °C using an electrical heater. The heater was designed in such a way that the cold finger of the cell was kept colder than the main reservoir.

The total internal reflection of the main laser beam from the interface of glass prism-Rb vapor was recorded by a photodetector and demodulated via a lock-in amplifier. Furthermore, an 88 mT external magnetic field was applied to the sample by inserting two neodymium magnets on the sides of the prism in appropriate directions to configure different geometries. Finally, the resultant data were recorded by a computer. During the measurement, all devices were synced through the LabView program. This applied magnetic field in theoretical calculation and also, experimental setup was set at 880 G (88 mT). Neodymium magnets with high coercivity and 7 × 13 × 5.5 cm dimensions were used and a Lakeshore gaussmeter (Model 425) was utilized to measure the magnetic field strength. The gaussmeter provided 1 mT measurement resolution and the inhomogeneity of the field over the 1 mm size of the laser beam was about 0.7 mT.

In the measurement, we set the 795 nm and 780 nm laser light to linear, right, and left circular polarizations consecutively and tuned the angle of incidence of laser light to approximately 0.05 degrees larger than the critical angle.

## Results and discussion

**Figure 3:**
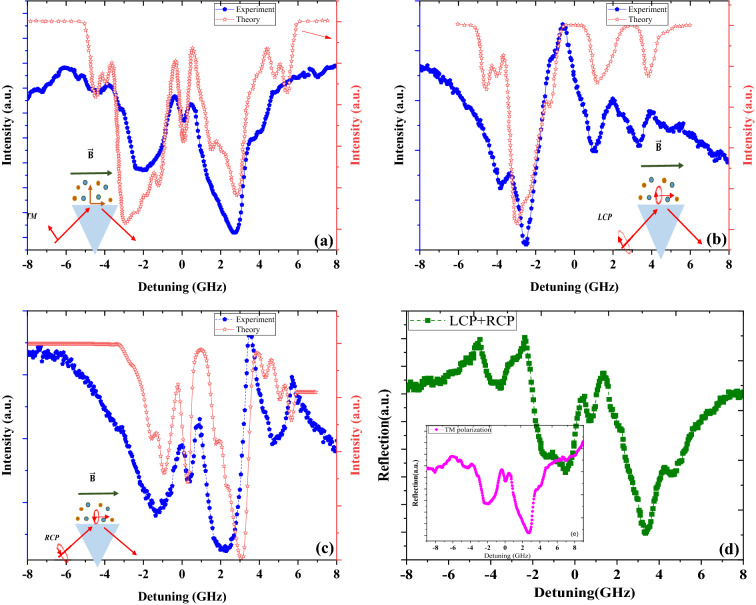
Comparison of experimental (top) and simulation (bottom) results for (**a**) absorption of evanescent waves formed by p-polarized incidence light, (**b**) absorption of evanescent waves formed by left circularly polarized light, (**c**) absorption of evanescent waves formed by right circularly polarized light, (**d**) summation of normalized LCP and RCP experimental data of (**b**,**d**), inset shows experimental data shown in (a), brought for comparison.

Consideration of the polarization plane and propagation direction of evanescent waves along the applied magnetic field (parallel to the prism surface) results in a Faraday configuration. This obviously contradicts the configuration of the magnetic field and free space laser light before reaching the prism (Fig. 2). The solution of the Maxwell equations for the Faraday configuration will result in right and left circular polarizations as normal modes. Thus, the allowed transition lines that can satisfy the selection rules of $\Delta m = \pm 1$ will be $\sigma^{ + }$ (left circularly polarized light) and $\sigma^{ - }$ (right circularly polarized light) transitions. We performed experimental measurements when the incoming light was right and left circularly polarized. Figure 3b,c show the experimental and simulation data together with very good agreement. Nevertheless, we can investigate changes in polarization by analyzing only experimental data for the first time. According to the Maxwell equation and the principle of normal modes, in the Faraday configuration, incoming light is decomposed into right and left circular polarizations, and the final absorption is the sum of the absorption of two normal modes with their specific share. As stated in the previous section, the polarizability tensor depends on transitions with $\Delta m = \pm 1$ and 0; thus, the right and left circular polarizations will only excite $\sigma^{ - }$ and $\sigma^{ + }$ transitions, respectively. It is concluded that the addition of absorption spectra of circular normal modes, with equal shares in the case of incoming p polarized light, must result in the absorption of incoming linearly polarized light. Figure 3d represents the result of adding normalized measured absorption of formed evanescent wave by incoming right and left circular polarized laser light.

Figure 3a presents the theoretical and experimental absorption of D1 line spectra of evanescent waves thorough anisotropic Rb vapor. It is apparent that there is good agreement between the two data points, as expected. The good agreement between the summed data and absorption under incoming linear p-polarized light shows that the state of polarization of the evanescent wave, excited by p-polarized light, will be linear when the angle of incidence of light equals the critical angle.

To evaluate our method, we predicted the polarization of evanescent waves from Eq. (1), when the effect of the refractive index of rubidium vapor on variations in the polarization of evanescent waves was neglected. This equation predicts the polarization of the evanescent wave at the critical angle as linearly polarized for incoming p-polarized light.

Therefore, the output of our method is in agreement with the polarization state predicted by the abovementioned relations (Eq. 1). Another direct and useful consequence of comparison of the experimental and simulation results is that it can be used to show variations of polarization of evanescent field from polarization of normal modes in any other complicated nanostructures, when excitation is done by incoming polarized light according to normal mode polarization state predicted by the theory. For example, we set the polarization state of free space incoming laser light to a circular polarization state as the normal mode of anisotropic atomic vapor.

A comparison between the experimental and simulation data in Fig. 3b,c shows that the total internal reflection of right or left circular polarized laser light will produce right or left circular polarized evanescent waves, respectively. Prediction of the state of polarization of evanescent waves formed by the total reflection of circularly polarized laser light from prism, with the help of Eq. (1) is not as straight as the prediction in the case of linearly polarized incoming light.

As the angle of incidence of light grows larger than the critical angle, the state of polarization of evanescent waves will become more complicated. As explained before in Eq. (1), when the incident light is TE polarized, the evanescent field is linearly polarized with the electric field perpendicular to the plane of incidence of light (Ey). However, in the case of TM-polarized light, the evanescent field is elliptically polarized with two electric field components in the x and z directions (Ex and Ez), which are 90° phase shifts relative to each other.

**Figure 4:**
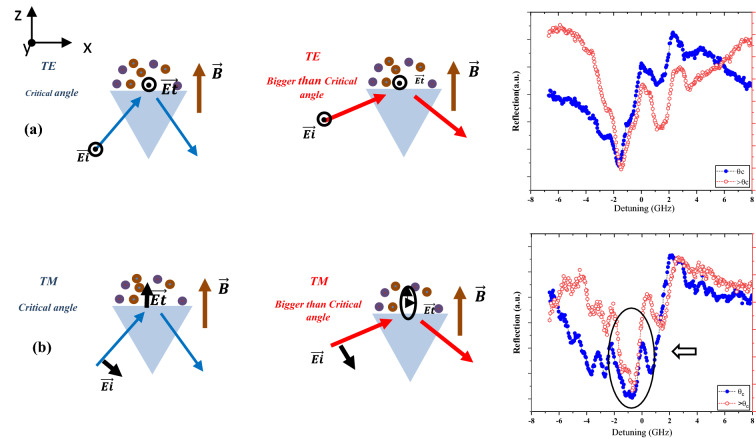
Results of evanescent wave reflection, (**a**) comparison of s polarization at a critical angle and 7 degrees larger than the critical angle, (**b**) comparison of p polarization at a critical angle and 7 degrees larger than the critical angle.

To confirm the ability of our method to trace the changes of polarization state of evanescent field, we change the Faraday to Voigt configuration, and the light source to D2 line. This change will increase the degrees of freedom as the direction of the electric field with respect to the magnetic field and propagation direction of the evanescent wave becomes important. Figure 4a,b compare the results for incoming p and s polarization at the critical angle in the comparison by angle of incidence larger than that.

From the results, it is clear that alteration of the angle of incidence in the case of p polarization changes the absorption spectra, but in the case of incoming s polarization, no change was observed (we focus on the frequency of absorption related to hyperfine transitions, and small changes in the intensity of absorption that are equally distributed in the detuning range are not our goal).

Therefore, although in both cases the incident light is linearly polarized, it is apparent that the effect of the change in the angle of incidence on the results is completely different, as shown by the arrow in Fig. 4b. Explanation of changes of spectra of evanescent wave formed by incoming s and p polarizations would be possible again based on Maxwell equations including differences of configurations and allowed atomic transitions in any configuration. In the case E||B, the only allowed transitions are π transitions, while in the case ${\text{E}} \bot {\text{B}}$, the only allowed transitions are $\sigma^{ \pm }$. According to this theory, evanescent waves resulting from the total internal reflection of p-polarized and s-polarized light at a critical angle will be absorbed by $\pi$ and $\sigma^{ \pm }$ transitions, respectively. A comparison of the absorption of two different incoming polarizations at two different angles shows that the spectrum of TE polarization does not change with increasing angle of incidence. Therefore, it is easy to conclude that the state of polarization of the evanescent wave formed by the total internal reflection of TE polarized light in angle over the critical angle is TE polarized, as predicted by theory.

A comparison of the spectra of the total internal reflection of TM polarized light clearly shows that the state of polarization of the evanescent wave by a change in the angle of incidence will not remain the same. As absorption depths are a result of atomic transitions, any changes in absorption spectra must be interpreted by changes in atomic transitions. The results clearly show that absorption of evanescent waves at angles larger than the critical angle has transitions in common with both p and s transitions. This means that the spectra are a result of all transitions with $\Delta m = \pm 1,0$. As the angle of incidence of p-polarized light grows, the polarization of the evanescent wave becomes elliptical, as predicted by Eq. This means that the plane of polarization is parallel to the plane of incidence of light. The semielliptically polarized evanescent field has components transverse and parallel to the magnetic field and all $\sigma^{ \pm }$ and $\pi$ transitions. Note that without anisotropic characteristics of rubidium vapor, changes in spectra with alteration of the incidence angle would not be observed, as there is no difference in the absorption of isotropic media in different directions of propagation of light. This property of atomic vapors makes them a good candidate to perform spectroscopic measurements as a candidate to trace any changes in the state of polarization of the near field by any external factor.

In addition to anisotropy, resolution for the detection of small changes requires a good degree of separation between absorbing atomic transitions. Applying an external magnetic field yields separation on absorbing transitions as described in Eq. (8) and during Hamiltonian.

In addition to anisotropy, the ability to detect small changes requires a resolved hyperfine transition spectrum. Applying an external magnetic field causes Zeeman splitting that increases resolution of detection of changes in absorption spectrum. The main factor that causes hyperfine structure to be not resolved is Doppler broadening. As the magnetic moment of alkali metal atoms are very similar, hyperfine splitting of atoms in specified applied magnetic fields mainly depends on Doppler broadening. Studies show a decrease in Doppler broadening with an increase of atomic number of alkali metal atoms. For example, amount of Doppler broadening at 373 K for ${}^{39}{\text{K}}$ is 862 while for ${}^{87}{\text{Rb}}$ is 559^41^. This makes rubidium metal vapor a better choice for polarization spectroscopy of evanescent waves under a smaller amount of magnetic field, compared to sodium or potassium. In this study, we investigated both D lines of rubidium metal to show that both D lines have suitable resolution to study the properties of evanescent fields in anisotropic media.

## Conclusions

Our results show that atomic spectroscopy of rubidium vapor under an applied magnetic field has enough sensitivity to small changes in the polarization state of evanescent waves if a suitable configuration is chosen. Additionally, we showed that simulation of the absorption spectra of the polarization state set to normal modes of an anisotropic atomic medium is a good standing point for comparison of experimental and simulation data with the purpose of sensitivity to any probable change in the polarization state of evanescent waves. We showed that when changes in the polarization of evanescent waves lead to changes in the geometrical configurations of the magnetic field and electric field of evanescent waves, significant changes in the absorption spectra will appear. Changes in the absorption spectra would be used to follow changes in the polarization of the propagating wave, as the results approve allowed transition lines that satisfy the selection rules of ${\Delta }m_{F} = \pm 1$ for $\sigma^{ + }$ (LCP) and $\sigma^{ - }$ (RCP) transitions in anisotropic Rb vapor. We changed the polarization of evanescent waves by changing the angle of incidence of laser light because the angle of incidence was set to the critical angle and larger than that, and the ellipticity of the polarization of evanescent waves changed for incoming p-polarized light. We sorted the necessary steps for predicting the polarization state of evanescent waves in an unknown nanostructure as (a) determining the normal modes of magneto-optical configurations from Maxwell equations, (b) utilizing the simulation process to predict the absorbance of detuned polarized waves in the desired configuration, and (c) comparing experimental data with simulation to show the share of any normal mode polarization state in the total polarization state of an arbitrary near field. Analyzing the absorption spectra of evanescent waves in more complicated nanostructures in Voigt and Faraday configurations will show us possible changes in the polarization states of evanescent waves due to confinement in nanostructures.

## Data Availability

Data underlying the results presented in this paper are not publicly available at this time but may be obtained from the authors upon reasonable request.
